# Comparison of the Ekblom-Bak Submaximal Test to a Maximal Test in a Cohort of Healthy Younger and Older Adults in the United States

**DOI:** 10.3389/fphys.2020.550285

**Published:** 2020-11-06

**Authors:** Stephanie A. Schultz, Jennifer Byers, Tammie L. S. Benzinger, Dominic Reeds, Andrei G. Vlassenko, W. Todd Cade, Manu S. Goyal

**Affiliations:** ^1^Mallinckrodt Institute of Radiology, Washington University School of Medicine, St. Louis, MO, United States; ^2^Center for Human Nutrition, Washington University School of Medicine, St. Louis, MO, United States; ^3^Program in Physical Therapy and Department of Medicine, Washington University School of Medicine, St. Louis, MO, United States

**Keywords:** submaximal test, cardiorespiratory fitness, cycle ergometer, older adults, feasibility

## Abstract

Cardiorespiratory fitness (CRF) is routinely investigated in diverse populations, including in older adults of varying physical activity levels. Commonly performed maximal exercise testing protocols might be contraindicated and/or inadequate for older individuals who have physical or cognitive impairment. Moreover, early termination of an attempted maximal exercise test could result in underestimation of CRF in this population. The goal of the current study was to compare CRF estimates using the Ekblom-Bak (EB) submaximal exercise test – previously validated in a cohort of Scandinavian adults – versus a subsequent maximal exercise test in a diverse, Midwestern United States cohort. Fifteen generally healthy individuals were included in this study who were either “Young” (25–34 years old) or “Older” (55–75 years old) as well as either sedentary or highly active. Participants completed the EB submaximal exercise test, followed immediately by a maximal exercise test. We found that all 15 individuals were able to successfully perform the EB submaximal testing method. Across the wide range of volumes of maximal oxygen consumption (VO_2_max; 12–52 ml/kg/min), the EB submaximal estimates of VO_2_max correlated highly with the maximal test based values (Pearson’s *r* = 0.98), but with a small bias (6 ml/kg/min, 95% limits of agreement −1.06 and −11.29). Our results suggest that the EB submaximal testing method may be useful in identifying wide differences in CRF among a diverse cohort of older adults in the United States, but larger studies will be needed to determine the degree of its accuracy and precision in identifying smaller differences.

## Introduction

Cardiorespiratory fitness (CRF) and physical activity are important physiological and behavioral measures that predict health outcomes throughout the lifespan. In adulthood, poor CRF and sedentary lifestyle are associated with risk for cardiovascular disease (Kodama et al., 2009; Khan et al., 2015; Laukkanen et al., 2019), Type 2 diabetes (Lavie et al., 2014; Zaccardi et al., 2015), and Alzheimer disease (Kurl et al., 2018; Silva et al., 2019).

Improvement of CRF, through engagement in regular physical activity, leads to a significant improvement in health outcomes including lowering risk of all-cause mortality (Harber et al., 2017), cardiovascular disease (Safdar and Mangi, 2020), and diabetes (Tuomilehto et al., 2001). There is now growing interest to consider the predictive value and effects of improved CRF on brain aging and dementia.

Assessment of maximal oxygen consumption (VO_2_max) during a graded exercise test is considered the gold standard measure for evaluating CRF (Kenney et al., 1995). Obtaining a true measure of VO_2_max, where a plateau in oxygen consumption is observed despite further increases in intensity of exercise, is often challenging in certain populations, including older adults, individuals with physical limitations, and with cognitive impairment. In at-risk individuals, maximal exercise testing requires immediate oversight by a trained physician and in some cases may not be feasible due to the increased risk of adverse events. Peak exercise testing also requires the tested individuals to give a high level of effort and understanding of the test in order to exercise to volitional exhaustion. Furthermore, measurement of VO_2_max testing requires expensive equipment for gas analyses, which is burdensome on the clinical and research teams to acquire and maintain. Importantly, all of these challenges might restrict cohorts that can participate in important studies relating CRF to health and cognitive outcomes.

Submaximal exercise testing is therefore commonly used to predict VO_2_max, as a proxy measure of CRF, particularly when laboratory equipment or physician supervision is unavailable or when the target population is unlikely to consistently achieve volitional exhaustion. Interest in the role of maintaining CRF throughout life to help protect against age-related diseases, such as Alzheimer disease, is rapidly increasing (Hamer and Chida, 2009; Pentikainen et al., 2019). The need to develop and examine the ability of submaximal exercise tests to be reliable in mixed population across a wide range of age, physical functioning, and cognitive functioning thus cannot be overstated.

Submaximal tests are typically based on heart rate response at one or more submaximal work rates and often utilize a regression-equation method to predict VO_2_max. A range of submaximal exercise testing protocols exist, each of which have optimal testing populations that demonstrate a range of reliability and predictability (correlation coefficients of 0.52–0.93) for maximal exercise testing based measures of VO_2_max (Noonan and Dean, 2000). Furthermore, some studies suggest that submaximal test based estimates of VO_2_max might be less accurate in African American versus white men (Vehrs and Fellingham, 2006).

A recently developed Ekblom-Bak (EB) cycle ergometer test (Ekblom-Bak et al., 2014; Bjorkman et al., 2016) for prediction of VO_2_max is low-risk, easy to administer, and shown to be valid for a wide range of aerobic capacities and ages (Vaisanen et al., 2020). The initially reported (Ekblom-Bak et al., 2014) association between estimated and observed VO_2_max using the EB method was *r* = 0.91, and showed significant improvements on corresponding coefficient of variation (9.3%) compared to the Åstrand-Rhyming method (18.0%). The EB test has been recently tested in an older population showing good agreement with maximal test VO_2_max estimates, which were further improved when applying the EB equation designed for women to both sexes (Vaisanen et al., 2020). While these results are highly promising for use of the EB method in aging studies, they were performed in a Scandinavian population and may or may not be replicable in other populations.

The goal of the current study was to compare CRF estimates using the EB submaximal exercise test – previously validated in a cohort of Scandinavian adults – versus a subsequent maximal exercise test in a diverse, Midwestern United States cohort of Young (25–34 years old) and Older (55–75 years old) individuals, and at different physical activity levels.

## Materials and Methods

### Participants

The Washington University in St. Louis Institutional Review Board approved all study procedures, and informed consent was obtained from all individual participants. Fifteen individuals recruited from a larger pilot study focused on CRF and brain metabolism were included in this small study based on completion of both submaximal EB and maximal exercise tests. Participants were screened over the phone by study team member (JB or SAS). Participant eligibility was based on age [either 25–34 years old (*n* = 4) or 55 years or older (*n* = 11)] and current self-reported physical activity levels on the International Physical Activity Questionnaire [IPAQ; either Sedentary (MET minutes/week < 1,000; *n* = 6) or Active (MET minutes/week > 3,000; *n* = 9; Booth, 2000]. Individuals were excluded from the study if they self-reported history of cardiovascular disease, Type 1 or Type 2 diabetes mellitus, body mass index > 35, and/or severe untreated hypertension (>200/100 mmHg). A full list of medical history questions assessed during phone screening is included in Supplementary Material. Further, a study physician reviewed a screening electrocardiogram (ECG) prior to the testing. This screening ECG was evaluated based on criteria adapted by the AHA Guidelines on Exercise Testing (Gibbons et al., 2002) to define criteria for reviewing the screening ECG. In particular, participants with: (1) evidence of pre-excitation, Wolff-Parkinson White (WPW), (2) resting ST depression >1 mm, (3) left bundle branch block (LBBB), or (4) evidence of prior silent myocardial infarction (MI; e.g., pathologic Q-waves) were excluded. To note, only one participant was excluded on the basis of ECG alone: a young participant with evidence of pre-excitation/possible WPW.

### Submaximal Exercise Test

Participants were instructed not to perform any heavy or prolonged physical activity the day before or on the day of the test. Participant body mass and height (Health o meter® Professional Scale, 500 kg, McCook, IL, United States) were obtained upon arrival to the testing center. The participants were informed of test procedures and equipped with 12-lead continuous ECG.

Tests were conducted on a recumbent cycle ergometer (Lode, Corival, Groningen, The Netherlands) with continuous 12-lead ECG monitoring (General Electric, Case, Milwaukee, WI, United States). Participants were instructed to pedal at a cadence of 60 revolutions per minute (RPM) for the duration of the test. Total duration of the test was 8 min, with an initial 4-min stage at a fixed work rate of 30 watts (W), directly followed by a higher individualized work rate that varied between 60 and 200 W for 4 min. The individualized work rate was subjectively chosen by the test leader (SAS) with regard to gender, age, training background, and current engagement in physical activity. Mean HR was recorded during the last minute of each work rate calculated as the average of the heart rate recorded at 3:15, 3:30, 3:45, and 4:00 minutes. Participants reported a Borg rating of perceived exertion (RPE) during both stages of the test, with the goal of reaching 13–15 during the second stage of testing.

VO_2_max was predicted using the EB prediction equation (Bjorkman et al., 2016) for women and men separately. For women, the equation used was

$$\begin{array}{l} \ln\;{\mathrm{VO}}_{2}\max=1.84390-0.00673\times \mathrm{age}\\ \;-0.62578\times \left(\Delta HR/\Delta PO\right)\\ \;+0.00175\times \left(\Delta PO\right)\\ \;-0.00471\times \mathrm{HR}\;\mathrm{at}\;\mathrm{initial work rate}, \end{array}$$

where the difference in HR between the high and initial work rate is denoted as ΔHR, and the difference in work rate (watts) between the high and initial work rate-stage is denoted as ∆PO. For men the equation used was

$$\begin{array}{l} \ln\;{\mathrm{VO}}_{2}\max=2.04900-0.00858\times \mathrm{age}\\ \;-0.90742\times \left(\Delta HR/\Delta PO\right)\\ \;+0.00178\times \Delta PO\\ \;-0.00290\times \mathrm{HR}\;\mathrm{at}\;\mathrm{initial work rate}. \end{array}$$

After entering the corresponding values into the equation, VO_2_max (in L min^−1^) was estimated by putting in the obtained value as an exponent in the natural logarithm.

### Maximal Exercise Test

Maximal exercise testing was performed on the same recumbent cycle ergometer (Lode, The Netherlands) with continuous 12-lead ECG monitoring following the submaximal test. Participants were allowed a short break of 5 min after the end of the submaximal exercise test. The maximal exercise testing procedure was standardized across all participants. All tests were done on the same bike ergometer with the same tester (SAS). Participants were instructed to pedal at a cadence of 60 RPM for the duration of the test. The work rate started between 20 and 30 W and increased by 20 W every minute until an RPE of 15 was reached, after which work rate was increased by 10 W every minute until volitional exhaustion. Continuous measurements of oxygen uptake (VO_2_), carbon dioxide production, and minute ventilation were obtained using a metabolic cart and two-way non-rebreathing valve (ParvoMedics TrueOne, Sandy, UT, United States). The system was calibrated prior to each test using standard gases with known concentrations and with a calibrated three-liter syringe. Maximum effort was determined based on the American College of Sports Medicine criteria (ACSM, 2014) that require meeting at least two of the following: (1) respiratory exchange ratio (RER) ≥1.1, (2) change in VO_2_ <200 ml with an increase in work, (3) RPE of 17 or greater, and (4) achieving at least 90% of age predicted maximal heart rate.

### Statistics

Pearson correlation coefficients were calculated between the estimated and measured VO_2_max L min^−1^and ml kg^−1^ min^−1^. To determine whether validity was different for different demographics, Pearson correlation coefficient for continuous demographic variables (VO_2_max and maximal HR) or *t*-test and Cohen’s d effect sizes for categorical demographic variables (age dichotomized, self-rated physical activity dichotomized, and gender) were used to compare the difference of estimated and measured VO_2_max ml kg^−1^ min^−1^ and demographic variables of interest. Age and self-rated physical activity were considered as dichotomous variables due to the bimodal distribution introduced by our recruitment criteria.

Lastly, while correlations test the relationship between estimated VO_2_max and measured VO_2_max, we additionally performed a Bland-Altman plot analysis to evaluate bias between the mean differences, and to estimate an agreement interval, within which 95% of the differences of the estimated VO_2_max, compared to the measured VO_2_max, fall. Limits of Agreement (LoA) were calculated using the equation: mean of the difference between estimated and measured VO_2_max ± 1.96 multiplied by the SD of difference between the measured and estimated VO_2_max. R software version 3.5.0 was used for the statistical analyses. Prior to statistical analyses, relevant model assumptions were checked with no violations detected.

## Results

Participant characteristics are presented in Table 1. The age and self-reported physical activity ranges for the participants were 25–73 years and 198–25,992 MET minutes per week, respectively. Due to our stringent entry criteria, our participants had few cardiovascular risk factors. No individuals in our sample had diabetes or used tobacco. All 15 individuals were able to fully tolerate and complete both the submaximal and maximal exercise tests and met ACSM criteria for maximal effort on the latter test.

**Table 1:** Background characteristics.

Characteristic	Range	Mean value (SD) or %(*n*)
Age, years		
Total	25–73	51.9 (16.5)
Young (*n* = 4)	25–28	26.5 (1.7)
Older (*n* = 11)	56–73	61.1 (5.5)
Body mass index	20–42	27.8 (5.9)
Female	–	60.0 (9)
White	–	80.0 (12)
HbA1c	4.4–6	5.2 (0.5)
Total cholesterol	111–268	192.9 (41.8)
HDL	37–112	74.6 (22.3)
LDL	50–163	101.2 (32.6)
Resting Sys/Dia BP	113–183/72–106	135.8 (21.0)/83.5 (10.0)
Self-report PA, MET-min/week		
Total	198–25,992	7,471 (8779)
Sedentary (*n* = 6)	198–900	499 (293)
Active (*n* = 9)	4,236–25,992	12,120 (8,605)
Submaximal exercise test		
Peak HR, BPM	103–179	137.0 (24.1)
Peak RPE	13–16	14.3 (0.9)
Estimated VO_2_max, L min^−1^	1.7–4.1	2.8 (0.8)
Estimated VO_2_max, ml kg^−-1^ min^−1^	17.3–58.7	36.9 (12.8)
Maximal exercise test		
Peak HR, BPM	129–206	166.5 (24.1)
Peak RPE	16–19	17.7 (1.0)
VO_2_max, L min^−1^	0.91–3.94	2.3 (0.8)
VO_2_max, ml kg^−1^ min^−1^	11.9–51.7	30.7 (13.2)

HbA1c, hemoglobin A1c; HDL, high-denisty lipoprotein; LDL, low-density lipoprotein; PA, physical activity; Sys, systolic; Dia, diastolic; BP, blood pressure; HR, heart rate; BPM, beats per minute; RPE, borg rating of perceived exertion; VO_2_max, volume of maximal oxygen consumption. Participants fasted >12 h prior to sample collection for HbA1c, total cholesterol, HDL, and LDL measurement.

**Figure 1:**
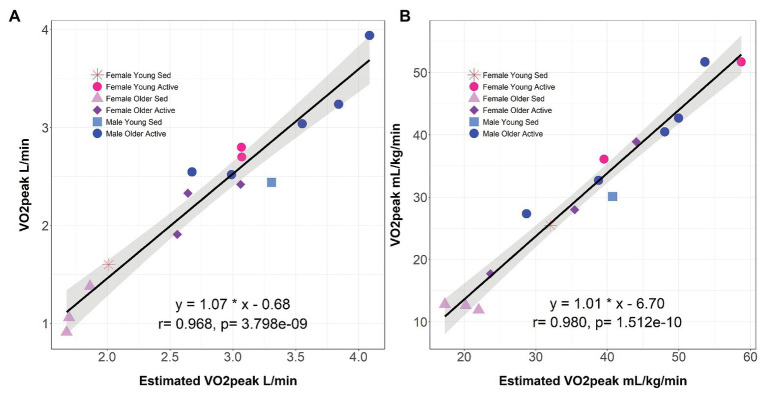
Correlation between estimated and measured VO_2_max. The submaximal Ekblom-Bak method estimate of VO_2_max compared to the measured maximal VO_2_max L min^−1^ **(A)** and ml kg^−1^ min^−1^ **(B)**. Linear regression equation for interpretation of intercept and slope are presented, where measured VO_2_max is the dependent variable and estimated VO_2_max is the independent variable. Pearson correlation coefficient and *p*-value are reported for each association examined. Shading represents 95% CI. Individuals are coded by gender, age (Young = 25–28 years old; Older = 56–73 years old), and current physical activity level (Sedentary = 198–900 MET-minutes/week; Active = 4,236–25,992 MET-minutes/week). VO_2_max, maximal oxygen consumption; Sed, Sedentary.

In our cohort with a wide range of activity levels, there was good agreement between the estimated VO_2_max by the EB test equation with respect to measured VO_2_max (*r* = 0.97, *p* = 3.80 × 10^−9^ and *r* = 0.98, *p* = 1.51 × 10^−10^ for L min^−1^ and ml kg^−1^ min^−1^, respectively; Figure 1).

The absolute difference between measured and estimated VO_2_max L min^−1^ were not associated with maximal HR (*r* = −0.30, *p* = 0.271), age [*d* (95% CI) = −0.03 (−1.3–1.2), *p* = 0.968], gender [*d* (95% CI) = −0.23 (−0.91–1.37), *p* = 0.705], nor VO_2_max L min^−1^ (*r* = −0.48, *p* = 0.07). Self-reported physical activity [d (95% CI) = 1.40 (−2.67 to −0.14), *p* = 0.019] was, however, associated with a difference between measured and estimated VO_2_max L min^−1^ levels. Specifically, Highly Active individuals showed a smaller mean difference between measured and estimated VO_2_max L min^−1^ levels of 0.38 L min^−1^ compared to Sedentary individuals (0.62 L min^−1^). This larger mean difference value in the Sedentary individuals was due to the estimated VO_2_max L min^−1^ levels being higher than the measured VO_2_max L min^−1^ levels.

**Figure 2:**
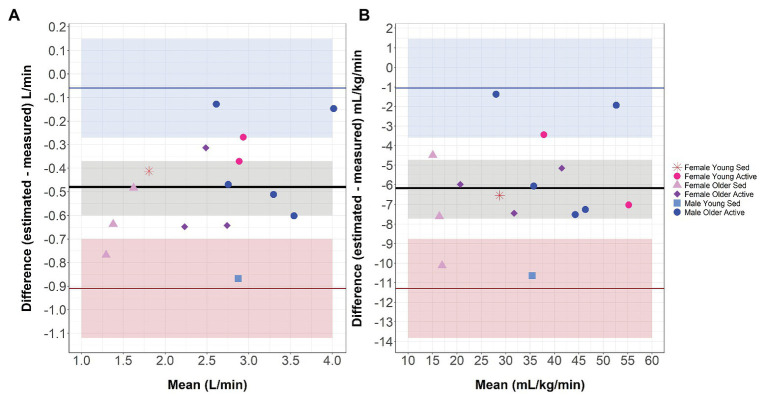
Bland-Altman Plot. Bland-Altman Analyses Plot between differences in estimated and measured VO_2_max compared to mean VO_2_max in L min^−1^ **(A)** and ml kg^−1^ min^−1^ **(B)**. Horizontal lines represent mean bias (black line), upper Limit of Agreement (LoA; blue line), lower LoA (red line). Shaded areas represent 95% CIs around mean bias (gray), upper COA (blue), and lower LoA (red). Individuals are coded by gender, age (Young = 25–28 years old; Older = 56–73 years old), and current physical activity level (Sedentary = 198–900 MET-minutes/week; Active = 4,236–25,992 MET-minutes/week). VO_2_max, maximal oxygen consumption; Sed, Sedentary.

As shown in Figure 2, the upper and lower LoA for L min^−1^ was −0.06 and −0.91, respectively, and for ml kg^−1^ min^−1^ was −1.06 and −11.29, respectively. The mean bias between the estimated and measured VO_2_max was −0.48 for L min^−1^ and −6.17 for ml kg^−1^ min^−1^.

## Discussion

The current study was aimed at examining the feasibility of the EB submaximal exercise test in a small diverse cohort typical of a Midwestern United States community, as well as to compare CRF estimates from the EB test versus a subsequent maximal exercise test in this cohort. We found that all 15 individuals were able to perform the EB submaximal testing method without issue, showing feasibility of this protocol in a diverse population. Importantly, we observed strong correlations between the EB method estimated VO_2_max values and the observed VO_2_max values, but with a small bias as observed with Bland-Altman Plot analyses. Our findings thus suggest that the EB-based estimate of VO_2_max is comparable to maximal exercise testing based VO_2_max in a representative United States cohort.

Prior submaximal testing protocols have only moderate validity in predicting VO_2_max, including a 12-min walk test (McGavin et al., 1976), 1-mile Rockport Fitness Test (Kline et al., 1987), and Åstrand and Ryhming Cycle Ergometer Test (Astrand and Ryhming, 1954), with reported correlations between measured and estimated VO_2_max of 0.52, 0.93, and 0.71, respectively (Astrand, 1960; McGavin et al., 1976; Kline et al., 1987). The Åstrand test is one of the most commonly used submaximal cycle ergometer tests and utilizes the heart rate response to one submaximal work rate. This test has been validated for a population up to only 65 years old. The current and prior studies now suggest that the EB method might be more comparable to maximal exercise testing across a wider age range.

However, similar to our findings that self-reported physical activity was associated with difference between measured and estimated VO_2_max L min^−1^ levels, prior studies (Bjorkman et al., 2016; Vaisanen et al., 2020) have found that individuals with lower CRF levels are more likely to have overestimated VO_2_max values with the EB method. One possible explanation for overestimation of VO_2_max values with the EB method in this study is that for safety and participant convenience reasons, the submaximal test was always performed before the maximal exercise test on the same day. It may be that individuals who have lower CRF and are less active are challenged more by performing two exercise tests in a row, compared to the high CRF and highly active individuals. Thus, the maximal exercise test based VO_2_max values might be underestimated in sedentary participants. However, we investigated this in our sample and found neither physical activity level nor CRF level (observed VO_2_max) was associated with percent of age-predicted HR (220-age) or RER at peak effort during maximal exercise test, suggesting that all participants performed similarly valid maximal exercise tests. As described in recent work (Poole and Jones, 2017; Schaun, 2017; Green and Askew, 2018), an alternative consideration may be that direct tests could also be underestimating the true value of VO_2_max in some individuals. Nonetheless, future validation studies might consider performing submaximal and maximal tests in varying order or on different days in individuals with a range of age and fitness levels.

These findings advance the existing body of literature suggesting that submaximal exercise tests, including the EB method, are a feasible method for estimation of VO_2_max and assessment of CRF across adulthood, genders, and physical activity levels. A limitation of this study is its small sample size, which preclude precise estimates of validity for subgroups within this cohort. Additionally, within the current cohort, we demonstrate the ability of the EB method to differentiate low or moderate CRF from high CRF. The validity of the EB method in a more narrow CRF range still remains to be determined. Future studies validating the EB method in the United States would be strengthened by a larger testing sample, longitudinal assessment, and an experimental structure where submaximal and maximal testing procedure order is performed both ways across age, gender, and physical activity level groupings. Future studies aimed at examining whether the relationship between the EB test VO_2_max estimates and maximal exercise test VO_2_ values differ as a function of cognitive functioning will be important for understanding the utility of the EB test in clinical populations with varying cognitive functioning.

The current study supports the notion that the EB method may be considered when designing research studies and clinical interventions aimed at evaluating CRF levels, especially in populations, where maximal exercise testing is not accessible due to equipment, physician supervision, or physical functioning constraints, or in populations that are not willing or able to perform maximal effort. Importantly, submaximal tests such as the EB method might be critical to avoid cohort effects in studies of aging. However, aside from identifying wide differences in CRF among individuals, further testing in the United States is required to determine the validity of the EB method in accurately distinguishing smaller differences in CRF.

## Data Availability Statement

The raw data supporting the conclusions of this article will be made available by the authors, without undue reservation.

## Ethics Statement

The Washington University in St. Louis Institutional Review Board approved all study procedures. The participants provided their written informed consent to participate in this study.

## Author Contributions

SS, DR, WC, and MG designed research. SS, JB, DR, AV, WC, and MG performed research. SS, WC, and MG analyzed data. SS, JB, TB, DR, AV, WC, and MG wrote and/or revised the paper. All authors contributed to the article and approved the submitted version.

### Conflict of Interest

The authors declare that the research was conducted in the absence of any commercial or financial relationships that could be construed as a potential conflict of interest.
