# Supplementary material for: Comparison of the Ekblom-Bak Submaximal Test to a Maximal Test in a Cohort of Healthy Younger and Older Adults in the United States
Source: Front Physiol. 2020 Nov 6;11:550285. doi: 10.3389/fphys.2020.550285 (PMC7677573; doi:10.3389/fphys.2020.550285)
Supplement: Supplementary file 1 [file Data_Sheet_1.pdf]

Study Screen

Age: ____________________ Height: _____________________ Weight: ___________________________

Currently employed: _______ Yes _______ No race______ sex ____ education level___________
DOB _________________________________

Injuries:____
Surgeries: ____
Hospitalizations: ____

Exercise Test Contraindications:

Y N Physical or health limitations that would prevent exercise, including riding a bicycle

Medical History:

Diabetes	Y	N	Controlled with Meds	Y	N	Type 1	Type 2
Coronary Artery Disease	Y	N	Controlled with Meds	Y	N		
Heart Attack	Y	N	Controlled with Meds	Y	N		
Bypass Graft Surgery	Y	N	Controlled with Meds	Y	N		
Cerebrovascular Disease	Y	N	Controlled with Meds	Y	N		
Peripheral Arterial Disease	Y	N	Controlled with Meds	Y	N		

Carotid Stenting	Y	N	Controlled with Meds	Y	N		
Congestive Heart Failure	Y	N	Controlled with Meds	Y	N		
Progressive Angina Pectoris	Y	N	Controlled with Meds	Y	N		
Nocturnal Pectoris	Y	N	Controlled with Meds	Y	N		
Severe or severe suspected stenosis	Y	N	Controlled with Meds	Y	N		
Acute Myocarditis	Y	N	Controlled with Meds	Y	N		
High Blood Pressure	Y	N	Controlled with Meds	Y	N		
Breathing Problems	Y	N	Describe				
Allergies	Y	N	Describe				
Head injury	Y	N	Describe				
Neurological Disorders	Y	N	Describe				
Concussion/ Loss of Consciousness	Y	N	Describe				

Heart Problems	Y	N	____________ ____	Liver/Kidney	Y	N	____________ ____
Cancer	Y	N	____________ ____	Thyroid/ Hormone	Y	N	____________ ____
Stroke	Y	N	____________ ____	Seizure/ Epilepsy	Y	N	____________ ____
Migraines	Y	N	____________ ____	Fainting	Y	N	____________ ____

Medications (including date started, dose/frequency/Schedule, and indication):

____

____

____

____

<table>
<tr><td colspan="3">Alcohol use:

_______________________</td><td></td><td>Tobacco use:

______________________________</td></tr>
<tr><td colspan="3">Drug use:

_________________________</td><td></td><td>Pregnant:

_________________________________</td></tr>
<tr><td colspan="2">Additional screening material</td><td colspan="3"></td></tr>
<tr><td>Y</td><td>N</td><td colspan="3">Administer PA assessment or confirm Actigraphy results. Is the participant sedentary or highly physically active?</td></tr>
</table>

Eligible: _______________________
